# Ictal Headache: A Case Report

**DOI:** 10.7759/cureus.99328

**Published:** 2025-12-15

**Authors:** Luis F Perez-Varela, Francisco Reyes-Salinas, Norma L Alvarado-Franco, Miguel A Del Rello-Diaz, Josue I Davila-Zarate

**Affiliations:** 1 Department of Internal Medicine, Hospital General de Zona #33, Instituto Mexicano del Seguro Social (IMSS), Monterrey, MEX

**Keywords:** electroencephalography, epilepsy, headache, migraine disorders, seizures

## Abstract

Migraine and epilepsy are traditionally considered to be unrelated conditions; however, they share pathophysiological mechanisms that could lead to headache associated with seizures or headache as the sole manifestation of epilepsy. We present a case of a 23-year-old female patient with severe headache as the only manifestation of epilepsy, and after multiple treatments without improvement, she underwent an electroencephalogram that showed epileptic activity coincident with the headache. The patient responded positively to oxcarbazepine, confirming the diagnosis of ictal headache. Ictal headache is an infrequent manifestation of epilepsy, primarily in focal seizures, and the diagnosis is often challenging due to the similarity of symptoms with other types of headaches and the difficulty in detecting epileptic discharges during the headache.

## Introduction

Conventionally, migraine and epilepsy have been identified as different entities; however, it has recently been documented that they could be different manifestations of the same pathophysiological process. The triggering mechanisms may overlap [1-3], resulting in headaches associated with epileptic seizures or epilepsy with headache as its only manifestation [4]. Based on this, different entities have been described: ictal headache, hemicrania epileptica, post-ictal headache, and migralepsy [1,4]. This implies a diagnostic difficulty as they present similar symptoms that may or may not be related, making it necessary to carry out complementary studies.

## Case presentation

A previously healthy 23-year-old woman was evaluated in an emergency department for loss of consciousness, apparently due to heat stroke. Her history revealed that the patient had symptoms of non-cardiac syncope. One month after that event, the patient came to our hospital with a pulsatile frontal headache of acute onset without triggers, which had been evolving over two weeks. The headache radiated to the left occipital region, was occasionally bifrontal, and progressively intensified, reaching 10/10 on the VAS about 30 minutes after onset. It was accompanied by phonophobia and photophobia with a fluctuating duration of 1 to 48 hours. Considering the severity of the headache and the previous syncope episode, she was admitted to the neurology department for further evaluation, where she was started on a treatment approach for her headache. 

She was previously treated with topiramate 50 mg daily and amitriptyline 25 mg daily as preventive treatment. She also admitted the use of NSAIDs, triptans, rimegepant, and tapentadol as abortive treatment; dosages were not provided at the time of the history-taking, and no improvement was reported with any of these treatments. Importantly, the patient mentioned that she was alternating medications and was not following any specific treatment routine. Her clinical history revealed no relevant family or psycho-social antecedents. 

On further evaluation, the migraine diagnosis was questioned due to no response to triptans and a poor correlation between headache and photophobia or phonophobia; the headaches were also not associated with physical activity. We started management for the secondary causes of headache, with paracetamol 1 g oral every 8 hours and zolmitriptan 2.5 mg oral if the pain persisted. Due to poor initial response, the patient was started on dexamethasone 4 mg IV daily for three doses. She was prescribed indomethacin 25 mg orally twice a day for three days, with just a partial response; she reported recurrence of pain within hours.

**Figure 1:**
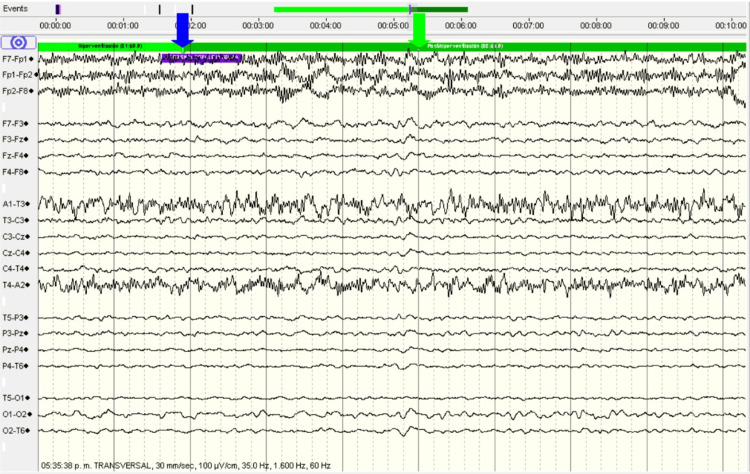
An EEG taken at the moment the headache started. A timestamp (blue arrow) shows when the patient indicated the onset of the headache, identifying a slow frequency pattern seconds later (green arrow). This study demonstrates an ictal-onset pattern correlating with headache onset. EEG: Electroencephalogram

**Figure 2:**
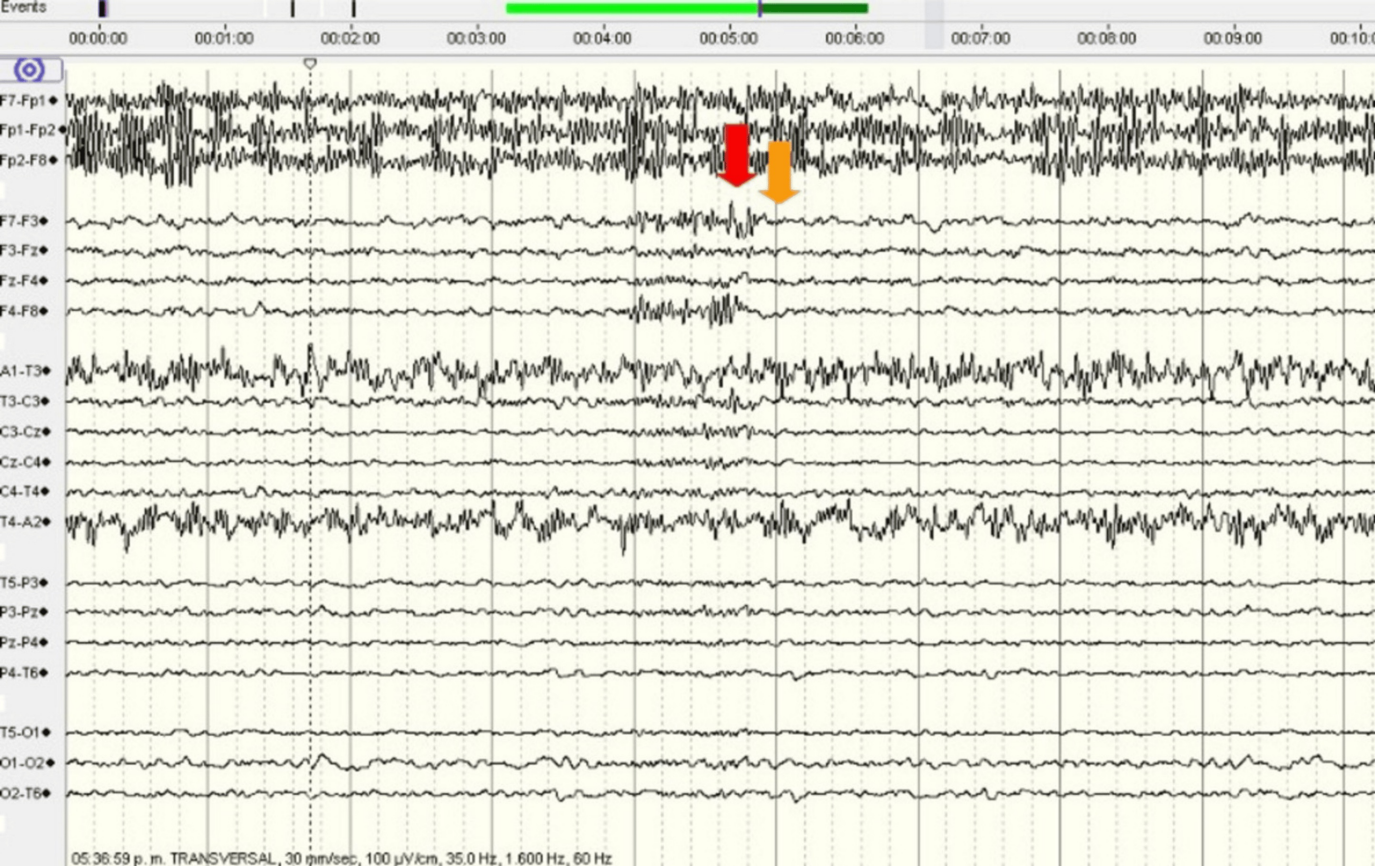
EEG continuation with ictal activity The red arrow indicates the presence of left frontal polyspikes with opposition-phase phenomenon at F7. The orange arrow indicates slow-frequency post-ictal waves.

During her evaluation, metabolic, infectious, and rheumatological causes were ruled out, with normal results for erythrocyte sedimentation rate (ESR), C-reactive protein (CRP), complete blood count, cholesterol, triglycerides, glycosylated hemoglobin, creatinine, serum electrolytes (including sodium, calcium, and magnesium), antinuclear antibodies (ANA), anti-neutrophil cytoplasmic antibodies (ANCA), and thyroid function. Later, we performed a lumbar puncture with normal results. A standard electroencephalogram (EEG) was requested as part of the diagnostic workup. Five minutes after the study began, the patient experienced an exacerbation of headache (Figure 1), and a few seconds later, slow, sharp waves were recorded at F3 and F7 on the left side. After six minutes, spikes in phase opposition appeared at F3, with left hemispheric propagation accompanied by posterior slowing (Figure 2). A brain CT scan and MRI with contrast were requested to complement the data documented in the EEG; results were normal, eliminating vascular or anatomic causes (i.e, intracranial mass/tumor, aneurysm, skull fractures, venous thrombosis). Based on these findings, ictal headache was suspected. Oxcarbazepine was initiated with remission of the headache, decreasing the dosage of analgesics. She was discharged after the improvement of the symptoms.

A month later, the patient arrived at the emergency department of our hospital. This time, she presented with disseminated dermatosis. During clinical history-taking, she reported that the condition had been evolving for three weeks. She was prescribed dexamethasone and loratadine; however, there was no response. A private dermatologist suspended oxcarbazepine due to suspicion of drug-induced dermatosis and restarted NSAIDs for pain control, with no response from the dermatosis or from the headache. The dermatology department of our hospital suspected a drug reaction with eosinophilia and systemic symptoms (DRESS) syndrome and performed a skin biopsy, which supported the diagnosis. During her second hospitalization, the patient had several headaches with the same characteristics as previously mentioned, apparently associated with the suspension of oxcarbazepine, requiring a new evaluation from the neurology department. Levetiracetam 500 mg oral every 24 hours was started, leading to improvement of the pain, and she was subsequently discharged after improvement of the dermatosis. In a follow-up consultation a month after the second hospitalization, the patient mentioned improvement of the symptoms and quality of life after initiation of levetiracetam (with only two episodes in the month), so the dose was increased to 500 mg twice daily. No more consultations are available in the electronic clinical record.

## Discussion

This condition was first described in 1980, when Lennox and Lennox introduced the term "migralepsy" as a combination of "migraine" and "epilepsy." However, it was not until 2012 that ictal headache was described as a headache that occurs at the same time as the onset of a focal epileptic discharge, which improves or resolves after the cessation of the seizure and is often ipsilateral to the epileptic focus. This definition also formed the basis of the first diagnostic criteria for the disease [2,5]. However, based on the latest update from the International League Against Epilepsy, we can classify it as a focal aware seizure, with painful somatosensory phenomena [2,6,7].

A clear pattern in clinical features has not yet been identified; however, it must include the manifestation of headaches, as well as migraine features with and without aura, hemicranial, and other associated symptoms [3,8]. It has been proposed that ictal headache is an isolated manifestation, lasting for seconds or days, with epileptiform discharges on the EEG that resolve immediately with the use of intravenous antiepileptics [4,5,8]. Additional hypotheses include that, unlike hemicrania epileptica, ictal headache can be ipsilateral or contralateral to epileptic discharge on an EEG and must be confirmed with epileptic discharge on an EEG and a positive response to intravenous antiepileptic treatment [1,4,8]. In addition, minor accompanying symptoms during the attack have been described in about half of the reported cases, including phonophobia and photophobia, nausea, pallor, dysarthria, agitation, and irritability [2,7]. 

The following diagnostic criteria were initially established: (1) headache of any characteristic; (2) evidence of an epileptic discharge on EEG that may be ipsilateral or contralateral to the headache; (3) it must be resolved immediately after the use of intravenous antiepileptic drugs [2].

Other authors propose that the diagnosis should be made in the context of a headache of any phenotype and the evidence of an epileptic discharge on EEG, without the third criterion, because it may be secondary to a lack of response to treatment [7], or that the seizure is so short that it is difficult to demonstrate response to treatment, and this will not necessarily exclude the diagnosis [4].

In 2018, the section "7.6: headaches attributed to epilepsy" was included in the ICHD-3, where ictal and post-ictal headaches are found. The diagnostic criteria [8] for this condition are any headache that meets the following conditions: (A) The headache must fulfill criterion C; (B) the patient must have a diagnosis of partial epilepsy; (C) there must be evidence that the headache develops simultaneously with a partial seizure and meets at least one of the following characteristics: (1) it is ipsilateral to the ictal discharge and/or (2) it remits or shows substantial improvement at the end of the ictal discharge. The headache should not meet the diagnostic criteria for another headache disorder as defined by the ICHD-3 [2,7].

Furthermore, the diagnosis of ictal headache may or may not be accompanied by other symptoms (motor, sensory, or autonomic), so to ascertain if it is a "pure" or "isolated" form of headache as the only manifestation of epilepsy, a differential diagnosis (criterion D) is required [3].

There is no specific treatment for ictal headache. The use of antiepileptics has been proposed, such as valproic acid and topiramate; other medications have been suggested, such as gabapentin, carbamazepine, oxcarbazepine, and zonisamide, although they have not been approved by the FDA to treat this condition [3,4]. They all share the characteristic of being classified as Class I drugs for focal onset seizures [9].

We consider this case to be representative of this type of pathological entity since it meets the criteria described above: headache lasting from minutes to days, ipsilateral or contralateral to epileptiform discharges on the EEG, and headache and EEG abnormalities resolve immediately after administration of antiepileptic drugs. This case demonstrates the importance of considering a headache of unusual presentation as a possible epileptic manifestation, especially when it doesn't respond to conventional treatment. As previously described, pure ictal headache is a rare diagnosis that could be underdiagnosed due to its criteria, which include evidence of epileptic discharge at the time of onset of the headache. It must also be considered that patients may often have an incomplete understanding of their disease; additionally, the use of antiepileptic drugs as a first-line prophylactic treatment for migraine is widespread, so the incidence of this condition could be higher than reported.

As our center is a limited-resource hospital, we did not have immediate access to benzodiazepines and experienced loss of follow-up; however, we present this as a representative case of ictal headache, particularly given the improvement after anticonvulsant treatment and worsening when it was discontinued due to DRESS syndrome, and only partial response to the initial low dose of levetiracetam.

## Conclusions

It is essential to consider ictal headache as a differential diagnosis in patients with headache with atypical characteristics (long duration, resistant to treatment, unusual symptoms), as its recognition and adequate treatment can considerably improve the patient's quality of life. To date, the literature regarding the approach and management of these patients is scarce, as this is a relatively recent diagnosis. More research is required to homogenize the definition, clinical presentation, diagnosis, and treatment.

This case is a reminder that not all headaches are the same, and every patient should undergo a complete evaluation of their condition, especially if it does not improve with conventional treatment. We also propose that ictal headache be considered as a potential diagnosis to help improve the quality of life for patients living with this condition who may not be receiving adequate treatment.
